# Historical data on cardiovascular health in Surinamese men

**DOI:** 10.1186/s13104-018-4030-1

**Published:** 2018-12-22

**Authors:** Lizzy M. Brewster, Jules Brewster

**Affiliations:** 1 Creatine Kinase Foundation, Amsterdam, The Netherlands; 2 Diaconessen Hospital, Paramaribo, Suriname

**Keywords:** Cardiovascular risk factors, Ethnic groups, Hypertension, Diabetes, Low and middle income countries

## Abstract

**Objectives:**

Cardiovascular risk factor burden was recently reported to be high in the Caribbean country Suriname. However, historical data for comparison were lacking. We report here rediscovered and hitherto unpublished aggregated data of what was apparently the first population study on measured blood pressure, diabetes, and cardiovascular health in men in this country, assessed in 1973. Data had been collected to raise the awareness of the local population for cardiovascular disease risk.

**Data description:**

We provide a table of the cardiovascular risk profile, including exercise, smoking, hypertension and diabetes of 243 Surinamese adult non-institutionalized men living in an urban setting in the capital Paramaribo in 1973. These data may help understand the cardiovascular risk factor escalation of the population in time as well as aid in projections of future cardiovascular disease in this middle income country.

## Objective

We report historical, hitherto unpublished data that had been collected in the capital Paramaribo, Suriname in 1973, to assess the cardiovascular health status in Surinamese men, in the “Have a Heart for your Heart” (HHH) study. The HHH study aimed at raising awareness of the local population for heart disease. Women had not been included because cardiovascular risk was perceived to be considerably higher in men. At the time of the study the population size of Paramaribo was around 150,000, with a national population of 320,000 citizens of whom 65% lived in an urban setting.

These data from 1973 might aid in the understanding of the high cardiovascular risk burden recently reported in the randomly selected urban citizens participating in the Healthy Life in Suriname (HeliSur) study [1, 2]. In this study in the population of Paramaribo (N = 250.000 citizens, with a national population of 560.000), a random sample (n = 1800) of citizens aged 18–70 years was drawn in 2013 (n = 1157 included, mean age 42 years; 37% men; 40% of African ancestry and 43% of South Asian ancestry) [1, 2].

In the HeliSur study, around 10% of the participants reported heavy, and 7% reported moderate regular leisure exercise (respectively 17 and 8% in men), 30% ever smoked tobacco (55% of the men), mean body mass index (kg/m^2^) was 27.8 (SEM 0.2), with 25.6 (0.2) in men; and 40% (41% of the men) had blood pressure levels higher than 140 mm Hg systolic or 90 mm Hg diastolic or were using antihypertensive drugs. Diabetes was found in 15% of the participants (13% in men), thus the risk factor burden in this urban, middle income country population was high. However, we had no previous data to assess time trends in risk factor prevalence [1, 2].

## Data description

The aim of the HHH study in 1973 had been to raise awareness for cardiovascular risk factors and cardiovascular disease. Men aged 30 years and older visiting the 15th national Surinade trade fair in Paramaribo in October 1973 had been requested oral informed consent to participate.

Participants had received information about cardiovascular disease from trained medical staff, and had filled out a questionnaire, including questions on physical exercise, smoking and self-reported diabetes. Furthermore, self-defined ethnicity was registered, and height, weight, and blood pressure had been measured. Hypertension was defined as blood pressure levels higher than 140 mm Hg systolic or 90 mm Hg diastolic, or using antihypertensive drugs. Participants with an abnormal cardiovascular risk profile were well informed regarding the medical care they needed and referred to the family doctor. Cardiologist Dr. J. Guda, who had supervised the data collection in 1973 is deceased. J. Brewster, one of the authors of this report, had collected the data, but these were never published. Only the recently rediscovered aggregated data are currently available (Table 1, Data file 1) [3].

**Table 1:** Overview of data files

Label	Name of data file/data set	File types (file extension)	Data repository and identifier
Data file 1	Selected cardiovascular risk factors in men, Paramaribo, Suriname	Word (.docx)	Figshare: 10.6084/m9.figshare.7203140 [3]

## Limitations

The limitations of these data are the inclusion of men visiting a national trade fair with the exclusion of women, and the limited data available. The main strength is the unicity of the data, as far as we know the only source of hitherto unpublished historical population data on the high cardiovascular disease risk burden in this middle income country.

## Abbreviations

HHH: Have a Heart for your Heart study
HeliSur: Healthy Life in Suriname study
SEM: standard error of the mean
